# Lightweight sCMOS-based high-density diffuse optical tomography

**DOI:** 10.1117/1.NPh.5.3.035006

**Published:** 2018-08-17

**Authors:** Karla M. Bergonzi, Tracy M. Burns-Yocum, Jonathan R. Bumstead, Elise M. Buckley, Patrick C. Mannion, Christopher H. Tracy, Eli Mennerick, Silvina L. Ferradal, Hamid Dehghani, Adam T. Eggebrecht, Joseph P. Culver

**Affiliations:** a Washington University in St. Louis, Department of Biomedical Engineering, St. Louis, Missouri, United States; b Washington University School of Medicine, Department of Radiology, St. Louis, Missouri, United States; c Boston Children’s Hospital, Fetal-Neonatal Neuroimaging and Developmental Science Center, Boston, Massachusetts, United States; d University of Birmingham, School of Computer Science, Birmingham, United Kingdom; e Washington University in St. Louis, Department of Physics, St. Louis, Missouri, United States

**Keywords:** diffuse optical tomography, functional neuroimaging, image reconstruction, visual stimulus, fiber optics, CMOS

## Abstract

Though optical imaging of human brain function is gaining momentum, widespread adoption is restricted in part by a tradeoff among cap wearability, field of view, and resolution. To increase coverage while maintaining functional magnetic resonance imaging (fMRI)-comparable image quality, optical systems require more fibers. However, these modifications drastically reduce the wearability of the imaging cap. The primary obstacle to optimizing wearability is cap weight, which is largely determined by fiber diameter. Smaller fibers collect less light and lead to challenges in obtaining adequate signal-to-noise ratio. Here, we report on a design that leverages the exquisite sensitivity of scientific CMOS cameras to use fibers with ∼30× smaller cross-sectional area than current high-density diffuse optical tomography (HD-DOT) systems. This superpixel sCMOS DOT (SP-DOT) system uses 200-μm-diameter fibers that facilitate a lightweight, wearable cap. We developed a superpixel algorithm with pixel binning and electronic noise subtraction to provide high dynamic range ($>10^{5}$), high frame rate (>6  Hz), and a low effective detectivity threshold ($∼200\;\mathrm{fW}/{\mathrm{Hz}}^{1/2}\text{-}{\mathrm{mm}}^{2}$), each comparable with previous HD-DOT systems. To assess system performance, we present retinotopic mapping of the visual cortex (n=5 subjects). SP-DOT offers a practical solution to providing a wearable, large field-of-view, and high-resolution optical neuroimaging system.

## 1 Introduction

Optical imaging has long held promise as a bedside neuroimaging technique. However, image quality has been a persistent challenge, particularly in comparison with the gold standard of functional brain imaging—functional magnetic resonance imaging (fMRI). Recently, high-density diffuse optical tomography (HD-DOT) has improved image quality dramatically.^1^^,^^2^ When matched within subjects against fMRI, HD-DOT now can obtain localization errors <5  mm and point spread functions with <15-mm full width at half maximum (FWHM), sufficient to localize functions to gyri.^3^ In addition to task activations, HD-DOT has been used for mapping distributed resting state functional connectivity (fcDOT) patterns. Although initial fcDOT reports were confined to sensory networks, such as visual and motor,^4^^,^^5^ recent results demonstrate the feasibility of mapping spatially distributed higher-level cognitive networks, including the dorsal attention and default mode networks.^2^

Despite these advances, HD-DOT has limited coverage and wearability due to mechanical challenges: increasing the coverage requires increasing the number of fibers, sources, and detectors, which results in heavier imaging caps and larger system sizes.^5^ Current large field-of-view HD-DOT caps, therefore, require significant infrastructure to support fiber weight in the cap. For routine and wide application, HD-DOT needs to become more wearable. One way to improve the wearability of the imaging cap is to reduce the fiber size; however, this directly reduces the amount of light transmitted to the detector. This poses a major challenge because HD-DOT requires low system noise and large dynamic range (e.g., $\mathrm{DNR}>10^{5}$) due to the high attenuation coefficients of blood in the brain and the need for optode separations >2  cm to sample the cortex.^1^ Useful metrics for quantifying the detector noise requirements for HD-DOT are the noise equivalent power (NEP) of the detector (e.g., NEP <20  fW/√Hz for a 3-mm detector) and detectivity of the system (e.g., $D=46\;\mathrm{fW}/\surd \mathrm{Hz}\text{-}{\mathrm{mm}}^{2}$), which are defined in Sec. 2.1.^6^

These performance requirements have led to discrete detector designs, generally avalanche photodiodes (APDs), as evidenced by the designs of current HD-DOT systems for imaging brain activity.^1^^,^^7^^–^^11^ A decrease in the fiber diameter in HD-DOT requires a detection scheme capable of achieving the same signal to noise ratio (SNR) and DNR specifications reported in previous systems with a significant decrease in photon flux. Here, we present a superpixel HD-DOT (SP-DOT) system that utilizes advancements in a megapixel CMOS sensor technology to overcome these technical challenges.

The reported SP-DOT enables HD-DOT imaging of brain activity with a lightweight cap. To compare specifications across multiple detectors, we discuss the concept of detectivity for optical neuroimaging systems and explore the effects from system parameters, such as frame rate, fiber size, and encoding strategy. System specifications and performance are tested with evaluations of raw data quality, via analyzes of the pulse waveform SNR in each channel, and with assessments of retinotopic mapping of visual activations in healthy adult volunteers, a benchmark for neuroimaging technologies. Collectively, these studies demonstrate the feasibility of SP-DOT and offer a practical solution to imaging brain activity using a lightweight wearable cap with HD-DOT spatial resolution.

## 2 Methods

### 2.1 Optical Neuroimaging System Specifications

To create a wearable, whole-head imaging system that weighs <1  lb, the commonly used 2.5-mm-diameter optical fibers need to be ∼10-fold smaller in diameter. Commercially available 200-μm-diameter optical fibers are closest to matching this requirement (NA=0.5, FP200URT, Thorlabs, New Jersey).^2^ However, the major challenge in redesigning HD-DOT with 200-μm-diameter fibers is the decrease in SNR that results from reducing the size of the fiber. To determine a detection design that enables the use of 200-μm-diameter fibers for HD-DOT, we begin by characterizing the light detection capabilities of APDs and CMOS sensors.

A useful metric for comparing these two detectors is detectivity, which is defined as the NEP divided by the area of the light collection

D=NEP/A, (1)

where D is the detectivity and A is the area. Because detectivity and NEP are typically defined for APD modules, our analysis also requires establishing these metrics for CMOS sensors. NEP relates the bandwidth of a signal measured by a photodetector to the optical power required to achieve an SNR equal to 1

$$P_{0}=\mathrm{NEP}\sqrt{\mathrm{BW}},$$ (2)

where $P_{0}$ is the optical power required for an SNR of 1 and BW is the bandwidth of the signal. Consider two systems each with equal illumination, which have a fiber collecting light from tissue and delivering the light to detectors with the same NEP. Assuming the area of the light on the detector is equal to the area of the fiber tip, these two systems will have different detectivities depending on the diameter of the fiber used to collect light. For example, the system that uses a fiber that is 10-fold smaller in area will have a 10-fold higher detectivity according to Eq. (1).

A detectivity of $∼100\;\mathrm{fW}/\surd \mathrm{Hz}\text{-}{\mathrm{mm}}^{2}$ is needed for high-quality HD-DOT.^2^ We compared the effective detectivity of six commercially available APDs to the superpixel sCMOS detection (Appendix B). The Hamamatsu C12703-01 had the lowest baseline NEP and the lowest effective detectivity with 200-μm-diameter fibers for HD-DOT (Table 2 and Appendix B). For this reason, we compared all the superpixel values with the C12703-01 APD module. Although the Hamamatsu C12703-01 module has the lowest detectivity of the detectors available on the market and is currently used for HD-DOT imaging with 2.5-mm-diameter optical fibers, the detectivity is still ∼70-fold too high for use with 200-μm-diameter fibers.^2^ Short source–detector separation measurements that sample the scalp and skull would have high SNR, but the longer separation measurements that sample the brain would have insufficient SNR. To measure these longer separation measurements, we require a detector with lower detectivity at a 200-μm-diameter collection.

Intriguingly, CMOS cameras have a 10,000-fold lower NEP at the single pixel level. Although the detectivity of a single pixel is sixfold higher than the APDs due to their small size, the fiber delivering light with 1:1 magnification will be a 200-μm-diameter circle on the sensor. Summing pixels within that 200-μm-diameter circle to create a “simple-binned” detector is predicted to match the requirements for HD-DOT imaging.

The rest of this section compares the CMOS characteristics to the commercially available APD module with the lowest NEP (Hamamatsu C12703-01) and is organized as follows: Sec. 2.1.1 explains the NEP, detectivity, and DNR specifications of the CMOS camera for individual pixels and simple-binned pixels at a standardized specification bandwidth of 1 Hz. Section 2.1.2 documents the 1-Hz specifications using a superpixel algorithm to sum the pixels while removing noise sources. Section 2.1.3 explores the effect of HD-DOT system parameters such as frame rate and encoding on the “effective” specifications of the superpixel system. Lastly, Sec. 2.1.4 details the specifications for the APD-based HD-DOT imaging system. All specifications discussed as follows are documented in Table 1.

**Table 1:** HD-DOT system specifications. Theoretical predictions and experimental measurements of specifications for HD-DOT at 830 nm. Effective NEP and detectivity for the 200-μm superpixel HD-DOT are calculated using 6-Hz frame rate, 25 encoding steps, and 1 wavelength. Effective specifications for the APD are calculated using 16 timesteps at 10 Hz. The area for simple-binned and superpixel calculations was determined by multiplying a single-pixel area by the number of pixels (N=697).

	Diameter or side (mm)	Area (${\mathrm{mm}}^{2}$)	DNR	NEP (fW/√Hz)	${\mathrm{NEP}}_{\mathrm{eff}}$ (fW/√Hz)	D ($\mathrm{fW}/{\mathrm{mm}}^{2}/\surd \mathrm{Hz}$)	$D_{\mathrm{eff}}$ ($\mathrm{fW}/{\mathrm{mm}}^{2}/\surd \mathrm{Hz}$)
HD-DOT requirements	—	—	$>1\times 10^{5}$	—	—	—	<100
APD (large fibers)	2.5	4.91	$1\times 10^{5}$	20	113	8.1	46.1
APD (small fibers)	0.2	0.031	$1\times 10^{5}$	20	113	1273	7202
Single pixel (theoretical)	$6.5\times 10^{-3}$	$4.23\times 10^{5}$	$1.2\times 10^{4}$	$2.2\times 10^{-3}$	0.2	52	4251
Simple binning and superpixel (theoretical)	0.2	0.029	$3.2\times 10^{5}$	0.058	4.7	2.0	161
Simple binning (experimental)	0.2	0.029	$1.5\times 10^{5}$	0.1	8.2	3.4	277
Superpixel (experimental)	0.2	0.029	$2.6\times 10^{5}$	0.07	5.7	2.4	194

#### 2.1.1 Pixel and simple-binning NEP, DNR, and detectivity at 1 Hz

The single-pixel NEP at 1 Hz (${\mathrm{NEP}}_{\mathrm{pix}}$) is calculated by relating the 1-Hz noise floor of the pixel to the optical power incident upon the pixel (Appendix A). Throughout our calculations, we assume that the primary noise source that contributes to the NEP of CMOS pixels is read-out noise. At 830 nm, the Zyla 5.5 sCMOS ${\mathrm{NEP}}_{\mathrm{pix}}$ is 0.0022  fW/√Hz, four orders of magnitude lower than the APD NEP of 20  fW/√Hz. By summing N pixels together, the NEP of the summed pixels (${\mathrm{NEP}}_{\text{sum}}$) is related to the NEP of a single pixel by

$${\mathrm{NEP}}_{\text{sum}}(N)=\sqrt{N}{\mathrm{NEP}}_{\mathrm{pix}}.$$ (3)

**Fig. 1:**
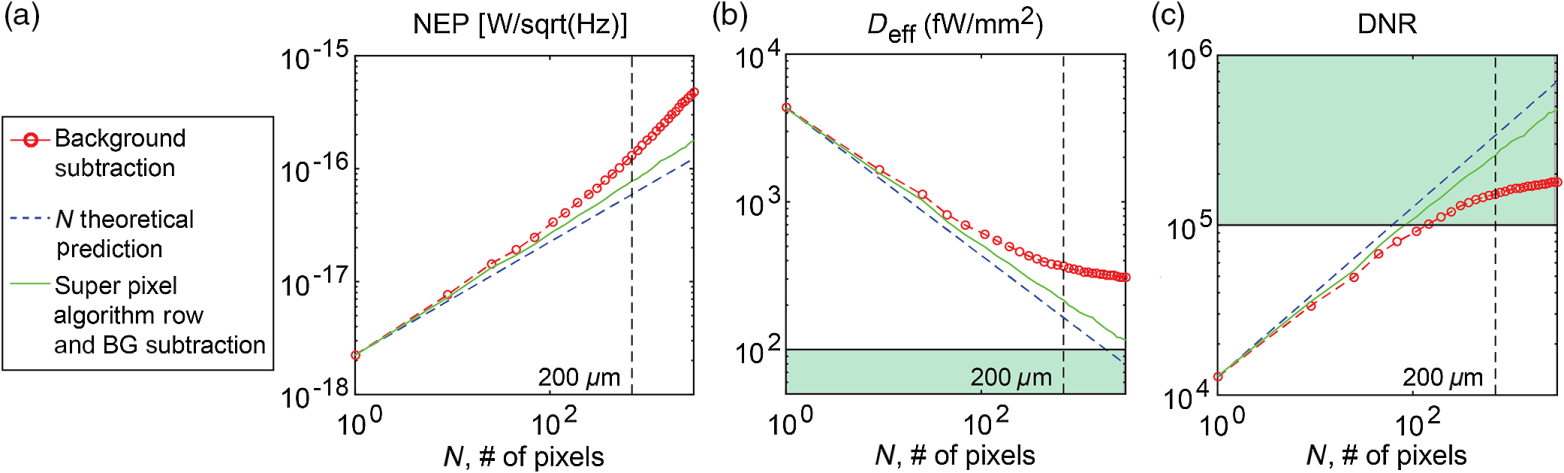
SP-DOT system specifications as a function of the superpixel size. (a) The NEP should increase as the square root of the number of pixels summed if the noise source is limited by read-noise (blue dashed line). Without subtracting off the correlated row noise (red line), the noise floor begins to deviate from the theoretical values at about 90 pixels. The size of the fiber tip on the CMOS sensor imaged at 1:1 magnification is 200  μm, denoted by the vertical dashed line. The (b) effective detectivity ($D_{\mathrm{eff}}$) and (c) DNR that are calculated based on the NEP are subsequently decreased and increased with larger N, respectively. After removing the correlated row noise with the superpixel algorithm (green line), the NEP, DNR, and $D_{\mathrm{eff}}$ are within a factor of 2 of the theoretical values. In addition, the DNR satisfies the required specifications for HD-DOT neuroimaging denoted by the green-shaded areas. The Deff value is 4× higher than what is required for HD-DOT but this can be accommodated by a 4× increase in light input.

The ${\mathrm{NEP}}_{\text{sum}}$ increases as the square root of N because the variance of the read-out noise increases by a factor of N [Fig. 1(a) and Appendix A]. Using Eq. (3) with N=697  pixels corresponding to the size of the optical fiber on the CMOS chip, the theoretical ${\mathrm{NEP}}_{\text{sum}}=0.058\;\mathrm{fW}/\surd \mathrm{Hz}$ (Table 1), ∼350-fold lower than the NEP of the C12703-01 APD modules (${\mathrm{NEP}}_{\mathrm{APD}}=20\;\mathrm{fW}/\surd \mathrm{Hz}$).

The 1-Hz detectivity of a pixel ($D_{\mathrm{pix}}$) is defined as the ${\mathrm{NEP}}_{\mathrm{pix}}$ divided by the area of the pixel ($A_{\mathrm{pix}}$). For 830 nm, the Zyla 5.5 sCMOS, $D_{\mathrm{pix}}=52\;\mathrm{fW}/\surd \mathrm{Hz}\text{-}{\mathrm{mm}}^{2}$, 24-fold lower than the detectivity of the APD system using 200-μm-diameter fibers ($D=1270\;\mathrm{fW}/\surd \mathrm{Hz}\text{-}{\mathrm{mm}}^{2}$).^2^ When summing pixels, the area increases linearly with the number of pixels summed. Combining this with, Eq. (3) provides the detectivity of summed pixels ($D_{\text{sum}}$)

$$D_{\text{sum}}(N)=\frac{\sqrt{N}{\mathrm{NEP}}_{\mathrm{pix}}}{NA_{\mathrm{pix}}}=\frac{D_{\mathrm{pix}}}{\sqrt{N}}.$$ (4)

Thus, the detectivity is inversely proportional to the $\sqrt{N}$ [Fig. 1(b)]. Using Eq. (4), the theoretical $D_{\text{sum}}$ for a 200-μm-diameter circle of pixels (N=697) is $2.0\;\mathrm{fW}/\surd \mathrm{Hz}\;\text{-}{\mathrm{mm}}^{2}$ (Table 1), 636-fold lower than the detectivity of the APD modules with 200-μm-diameter fibers ($D_{\mathrm{APD}}=1273\;\mathrm{fW}/\surd \mathrm{Hz}\text{-}{\mathrm{mm}}^{2}$).

The single-pixel DNR (${\mathrm{DNR}}_{\mathrm{pix}}$) is defined as the full well of the pixel (w) divided by the noise floor (σ). A single pixel has a ${\mathrm{DNR}}_{\mathrm{pix}}$ of $1.2\times 10^{4}$, two orders of magnitude lower than the APD and one order of magnitude too low for HD-DOT imaging. As the full well increases linearly with the number of pixels summed and the noise floor increases as the square root, the ${\mathrm{DNR}}_{\text{sum}}$ increases as the square root of the number of pixels summed [Fig. 1(c)]

$${\mathrm{DNR}}_{\text{sum}}(N)=\frac{Nw}{\sqrt{N}\sigma }=\sqrt{N}{\mathrm{DNR}}_{\mathrm{pix}}.$$ (5)

Using Eq. (5), the theoretical ${\mathrm{DNR}}_{\text{sum}}$ for a 200-μm-diameter circle of pixels on the sCMOS (N=697) is $3.2\times 10^{5}$ (Table 1), approximately threefold higher than the required DNR for HD-DOT.^2^

To test Eqs. (3)–(5) experimentally, we measured the specifications for a sCMOS sensor (Zyla 5.5, Andor Technologies) using a 200-μm-diameter fiber relayed to the CMOS sensor using 1∶1 magnification (Table 1). By experimentally summing N=697  pixels (6.5  μm width×6.5  μm height pixels) in a circular shape that corresponded to the 200-μm-diameter optical fiber, we were able to measure the NEP of summed pixels and therefore calculate the summed pixel detectivity and DNR. The detectivity of the summed pixels is a factor of ∼2 higher than the theoretical values (experimental $D_{\text{sum}}=3.4\;\mathrm{fW}/\surd \mathrm{Hz}\text{-}{\mathrm{mm}}^{2}$ versus theoretical $D_{\text{sum}}=2.0\;\mathrm{fW}/\surd \mathrm{Hz}\text{-}{\mathrm{mm}}^{2}$). Similarly, the DNR of the summed pixels is a factor of ∼2 lower than the theoretical values (experimental ${\mathrm{DNR}}_{\text{sum}}=1.5\times 10^{5}$ versus theoretical ${\mathrm{DNR}}_{\text{sum}}=3.2\times 10^{5}$). The discrepancy between theoretical and experimental values of NEP and DNR is exacerbated at large fiber diameters (Fig. 1), limiting the use of fibers to below ∼100  μm. To use 200-μm-diameter optical fibers, we developed a superpixel referencing algorithm to improve the detectivity and DNR of the CMOS sensor.

#### 2.1.2 Superpixel NEP, detectivity, and DNR at 1 Hz

**Fig. 2:**
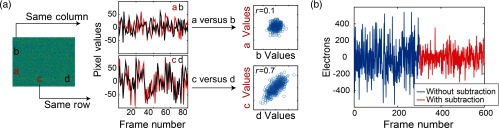
Correlated row noise. (a) Due to the readout structure of the sCMOS cameras, pixels within the same row have a correlated offset value after each readout. This can be demonstrated by plotting the values of two pixels as a function of time, which are either within the same column (pixels a and b) or same row (pixels c and d). The values of pixels a and b are not temporally correlated (r=0.1). The values of pixels c and d are highly correlated over time (r=0.7). (b) This correlated row noise can be removed by subtracting an average of multiple pixels within a row, reducing the temporal noise by 2×.

Although simple-binning of pixels improves the CMOS sensor’s detectivity and DNR, our experimental measurements of detectivity and DNR were worse than our theoretical predictions (Table 1 and Fig. 1). This mismatch between experimental results and Eqs. (3)–(5) was primarily caused by structured, non-Gaussian noise on the CMOS sensor. In addition to the shot-noise measured across the entire sensor (e.g., as measured in a background image), there is correlated noise within each row of the CMOS sensor caused by a voltage offset after each row readout [Fig. 2(a)]. Upon removal of this row noise, the temporal noise decreases by a factor of 2 [Fig. 2(b)].

**Fig. 3:**
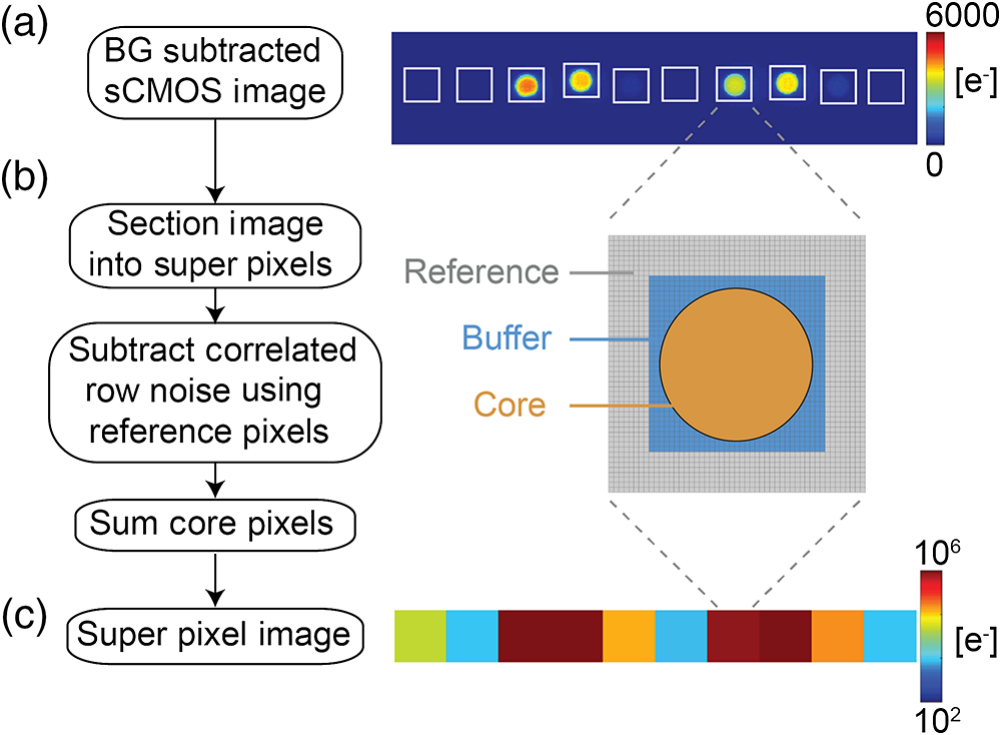
Superpixel algorithm. (a) A single raw sCMOS image of 10 detector fiber tips is background subtracted. (b) The image is partitioned into super pixels around each fiber tip and the values of the reference pixels are used for calculating the correlated row noise. (c) After subtracting the row noise, all core values are summed to create the final superpixel value per detector.

We calculated and removed the correlated row noise using a “superpixel algorithm.” The CMOS image of each fiber tip [Fig. 3(a)] was segmented into a core, buffer, and reference region using a system-specific mask [Fig. 3(b)]. Although the fiber tips required segmentation, the fiber tip locations and sizes did not change from experiment to experiment and thus the same segmentation mask was used run to run and day to day. The pixels segmented as the core regions are all pixels within a 100-μm-radius circle around a manually designated center pixel. This method based on distances ensured that the same number of pixels was summed for all fiber tips (N=697  pixels). The core region corresponds to the image of the fiber tip, the reference region provides measurements for determining the correlated row noise, and the buffer region serves to avoid any optical or electronic crosstalk from the core pixels into the reference region.

The row noise was removed using the following algorithm. Per row (j), the row noise ($R_{j}$) is defined by averaging the pixel values in the reference region of that row. To remove the row noise, we needed to scale the row noise by the number of pixels (N) in that row within the core. Thus, the scaled row noise is $N*R_{j}$. The superpixel value per row of the core ($SP_{j}$) is

$$SP_{j}=\overset{N}{\underset{i=1}{\sum }}(C_{i})-NR_{j}.$$ (6)

Note that because the fiber tip is circular, the number of pixels summed (N) for the core varies per row. The total superpixel value for a single fiber tip is then the sum of all j rows of the core

$$SP_{\text{total}}=\overset{J}{\underset{j=1}{\sum }}SP_{j}.$$ (7)

After applying the superpixel algorithm, a single CMOS image of 10 fiber tips is condensed into 10 superpixel values [Fig. 3(c)].

After removing these noise structures from the CMOS images, the noise [Fig. 2(b)], and therefore the NEP and detectivity of the 200-μm-diameter circle superpixel are decreased by ∼2× compared with simply summing pixels. The DNR increased approximately twofold using the superpixel algorithm. Now, the DNR ($2.6\times 10^{5}$) and detectivity ($D=2.4\;\mathrm{fW}/\surd \mathrm{Hz}\text{-}{\mathrm{mm}}^{2}$) are comparable with the same specifications of the APD using 2.5-mm-diameter optical fibers with 50% packing fractions, and closely match our theoretical predictions. Thus, with the superpixel summing algorithm, the DNR and detectivity for sCMOS detectors are promising for use in HD-DOT imaging when quoted at a 1-Hz bandwidth.

#### 2.1.3 Superpixel encoding: effective NEP and detectivity

HD-DOT requires higher data collection rates for temporal encoding of sources and to prevent sampling artifacts of biological signals. Therefore, the relatively slow data collection rates of CMOS cameras present challenges for successfully collecting the required amount of data for HD-DOT.^1^ All superpixel detectors collect light while a single source illuminates the tissue, which results in a single CMOS image acquisition for each source required for temporal encoding. To keep an overall “DOT frame rate” of the industry standard of 1 Hz, all K temporal encoding steps (number of source positions) are acquired in 1 s. As each temporal encoding step contains unique source–detector pair information, the amount of light collected per second is split evenly among all steps. The number of unique temporal steps, therefore, divides the flux into K different exposures. Additionally, each source is only turned on for a fraction of each temporal step, which is specified by the duty cycle (d).

Although there are multiple ways to account for this temporal encoding effect, here we assume a maximum illumination level from the sources. We therefore treat decreases in flux as effectively increasing the noise floor, thereby increasing the NEP. Because this treatment of NEP is a measure of the system, we are calling this value an effective NEP (${\mathrm{NEP}}_{\mathrm{eff}\_\mathrm{SP}}$). ${\mathrm{NEP}}_{\mathrm{eff}\_\mathrm{SP}}$ increases by a factor of K/d in comparison with the NEP of a single pixel because the photon flux decreases by a factor of K/d while the read-out noise remains the same

$${\mathrm{NEP}}_{\mathrm{eff}\_\mathrm{SP}}(N,K,d)=\frac{K\sqrt{N}}{d}{\mathrm{NEP}}_{\mathrm{pix}}.$$ (8)

Data collection frame rate is typically collected faster than 1 Hz to improve signal detection and avoid aliasing of hemodynamic signals. For example, to properly sample heart rate that typically ranges between 50 and 80 beats per minute during quiet rest HD-DOT data should be collected at or above 2 Hz. This means that all K temporal encoding steps need to be collected at least twice in 1 s. The number of times that all K temporal encoding steps need to be collected will be termed the “DOT sampling rate” of the system. Therefore, for a DOT sampling rate of f Hz, f*K total CMOS images will be collected. After sampling the entire encoding cycle at f Hz, the data will be low-pass filtered and temporally resampled back down to 1 Hz. Because the data are resampled, the total flux per DOT frame does not change with f; however, the number of reads per pixel changes (i.e., the variance of the read-out noise increases by a factor of f). The effective NEP is therefore given as

$${\mathrm{NEP}}_{\mathrm{eff}\_\mathrm{SP}}(N,K,d,f)=\frac{K\sqrt{fN}}{d}{\mathrm{NEP}}_{\mathrm{pix}}.$$ (9)

The resulting “DOT NEP” of the sCMOS 200-μm superpixel HD-DOT system at 6 Hz, 75% duty cycle, 25 encoding steps is experimentally measured to be ${\mathrm{NEP}}_{\mathrm{eff}\_\mathrm{SP}}=5.7\;\mathrm{fW}/\surd \mathrm{Hz}$ (Table 1).

As the detectivity is defined as the NEP divided by the collection area, the effective superpixel detectivity including all the parameters to run the HD-DOT system is

$$D_{\mathrm{eff}\_\mathrm{SP}}(N,K,d,f)=\frac{K}{d}\sqrt{\frac{f}{N}}D_{\mathrm{pix}}.$$ (10)

The APD system with 2.5-mm-diameter fibers and 50% packing fraction produces high-SNR HD-DOT data because the APD’s effective detectivity is $D_{\mathrm{eff}}=46\;\mathrm{fW}/\surd \mathrm{Hz}\text{-}{\mathrm{mm}}^{2}$ (Table 1). We, therefore, require the HD-DOT CMOS $D_{\mathrm{eff}}$ to be comparable or lower than that of the APD. With the superpixel algorithm, the Zyla 5.5 CMOS yields $D_{\mathrm{eff}}=194\;\mathrm{fW}/\surd \mathrm{Hz}\text{-}{\mathrm{mm}}^{2}$ for a 200-μm-diameter fiber [Fig. 1(c)], predicting that the CMOS camera would have fourfold lower $D_{\mathrm{eff}}$ than the APD system if the same amount of light was used. We can compensate for this factor of 4× using laser diodes to increase the light level by fourfold, allowable by ANSI limits. In comparison, the $D_{\mathrm{eff}}$ of the HD-DOT APD system using 200-μm-diameter fibers instead of 2.5 mm is $7202\;\mathrm{fW}/\surd \mathrm{Hz}\;{\mathrm{mm}}^{2}$, two orders of magnitude are too high for neuroimaging even with increased illumination to the ANSI limit. Comparing the effective detectivity of commercially available APD modules and the superpixel system shows that while the Hamamatsu C12703-01 has the lowest detectivity out of the APD modules, the superpixel system has ∼40-fold lower detectivity (200-μm Hamamatsu C12703-01 $D_{\mathrm{eff}}=7202\;\mathrm{fW}/\surd \mathrm{Hz}\text{-}{\mathrm{mm}}^{2}$ versus superpixel $D_{\mathrm{eff}}=194\;\mathrm{fW}/\surd \mathrm{Hz}\text{-}{\mathrm{mm}}^{2}$).

#### 2.1.4 APD NEP and detectivity

The signal generated by APD detectors is fundamentally different than CMOS cameras, and therefore we must derive expressions for the effective NEP and detectivity of APDs, which accounts for the frame rate, number of encoding steps (K = number of source positions), and duty cycle. The effective NEP of APDs is with reference to the NEP provided by manufacturers, which is quoted at 1 Hz (${\mathrm{NEP}}_{\mathrm{APD}\_1\;\mathrm{Hz}}$).

In contrast to CMOS cameras, the NEP of APDs is dependent on shot noise. The signal collected by the APD is proportional to the collection time, which decreases by a factor of K/d, resulting in the NEP increasing by a factor of $\sqrt{K/d}$. Due to the analog APD detector module, there is no added noise that results from increasing the frame rate if the data are downsampled to 1 Hz after collection (i.e., the effective APD NEP is independent of f). Therefore, the overall NEP for an APD HD-DOT system is given as

$${\mathrm{NEP}}_{\mathrm{eff}\_\mathrm{APD}}(K,d)=\sqrt{\frac{K}{d}}{\mathrm{NEP}}_{\mathrm{APD}\_1\;\mathrm{Hz}}.$$ (11)

The resulting effective “DOT NEP” of the APD 2.5-mm-diameter HD-DOT system at 10 Hz, 16 encoding steps, 50% duty cycle, is 113  fW/√Hz (Table 1).

The effective detectivity of the APD ($D_{\mathrm{eff}\_\mathrm{APD}}$) is calculated in the same manner as the CMOS: the 1-Hz detectivity ($D_{\mathrm{APD}\_1\;\mathrm{Hz}}$) is multiplied by the effects of encoding. Because we are calculating the effective detectivity of the APD HD-DOT system, the area needs to incorporate the packing fraction of the fiber bundles. The $D_{\mathrm{eff}\_\mathrm{APD}}$ accounting for all the parameters to run the HD-DOT system is

$$D_{\mathrm{eff}\_\mathrm{APD}}(f,K,d)=\sqrt{\frac{K}{d}}D_{\mathrm{APD}\_1\;\mathrm{Hz}}.$$ (12)

The resulting effective “DOT detectivity” of the APD 2.5-mm-diameter HD-DOT system at 10 Hz, 16 temporal encoding steps, and 50% duty cycle, is $D_{\mathrm{eff}\_\mathrm{APD}}=46\;\mathrm{fW}/\surd \mathrm{Hz}\text{-}{\mathrm{mm}}^{2}$ (Table 1).

### 2.2 Superpixel DOT System

To establish the feasibility of SP-DOT, we developed a 24-source and 28-detector system using a Zyla 5.5 sCMOS. The sources and detectors are located over the visual cortex to test the system performance with retinotopic mapping. This retinotopy system provides 264 usable source–detector measurements with a cap that weighs <1  lbs.

**Fig. 4:**
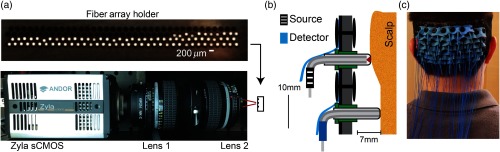
Superpixel HD-DOT system components. (a) The detector fibers terminate in an aluminum fiber array holder with the fibers separated by 200  μm (top). To take advantage of the readout speed of an sCMOS, the fibers are in rows of 36. The fiber array holder is imaged with a 1:1 magnification via a two-lens system (bottom). (b) The cap has bent fiber tips protruding through it that can slide in and out of the cap to match the curvature of the head. The source fibers have on average 2 mm of space to allow for the beam to expand for ANSI compliance. The detector fibers are flush with the scalp. Rubber stripping is used on the outside of the cap to provide a force toward the center of the cap. (c) The superpixel imaging cap consists of 24 sources and 28 detectors that cover the visual cortex. Due to the low weight of the fibers, the subject can support the cap and move around freely.

To relay the light from the detector fibers to the sCMOS chip, we designed dedicated detector boxes. The SMA end of the detector fibers screws into female SMA adapters on the custom-designed light-tight boxes. Inside the box, 0.75-m length 200-μm-diameter fibers (NA=0.5, FP200URT, Thorlabs, New Jersey) transmit light from the detector fibers to a dedicated aluminum block containing the cleaved fiber tips [Fig. 4(a)]. A 4f relay (consisting of two Nikon 50 mm f/1.2 Nikkor Ai-S Manual lenses) provides a 1∶1 magnification of the cleaved fiber tips onto the Zyla 5.5 sCMOS chip (Andor, Belfast, UK). To take advantage of the horizontal readout speed of the sCMOS camera, the 28 detector fiber tips were positioned horizontally in the fiber array. The fibers were separated by 200  μm of aluminum to minimize optical crosstalk on the sCMOS chip, resulting in rows of 23 detectors.

To optimize the amount of light reaching the head, we used laser diode sources (50 mW, 830 nm HL8338MG, 690 nm HL6750MG Thorlabs, New Jersey). The current delivered to the lasers was modulated by dedicated, high-bandwidth (20 MHz) digital input/output lines (PCI-6534; National Instruments, Austin, Texas). A single-source box contains 32 laser diode source channels and electronics in one standard 19-in. rack unit (height=1 RU or 1.75 in.). To focus as much light as possible onto the 200-μm-diameter fibers (NA 0.5, FP200URT, Thorlabs, New Jersey), a custom-designed, three-dimensional (3-D) printed laser coupler was attached to the source box containing a 2.5-mm ball lens (N-BK7, Edmunds Optics) placed between the diode and fiber SMA tip (SMA905, 10230A, Thorlabs, New Jersey). The laser coupler transmitted 60% of the laser light into the fiber.

To improve the fiber coupling, ergonomics, and comfort of the imaging system cap, we bent the fibers into a 90-deg curve just before entering the cap [Fig. 4(b)]. Both source and detector fibers had an SMA905 tip on one end and a bent tip on the other to sit in the cap. All right-angle tips were custom-made and polished. To maintain rigidity within the cap, a clear plastic sleeve was glued around the fiber tip that passed through the cap. To provide a safe and comfortable surface that sits against the scalp, biocompatible, translucent epoxy (UV10MED, Masterbond, New Jersey) covered the tip of the fiber [Fig. 4(b)]. An average of 2 mm of the clear epoxy was placed on the tip of the fibers to allow for the light to expand to a beam diameter of 2 mm once entering the skin ($0.2\;\mathrm{mW}/{\mathrm{mm}}^{2}$). Detector fibers had the minimum amount of epoxy needed to sufficiently cover the tip, averaging <0.2  mm. In total, the retinotopy SP-DOT system consisted of 76, 5-m-long optical fibers with an SMA connector on one end and a 90-deg curve on the other.

The imaging cap determines the spatial location of the source and detector fibers and maintains the coupling between the fibers and the scalp [Fig. 4(c)]. To provide the general shape of the cap, the outermost layer of the cap is composed of rigid thermoplastic molded to an anatomically correct adult-sized head model. A layer of soft neoprene on the inside of the thermoplastic provides comfort and supports the fibers. The array of sources and detectors is placed in two interlaced rectangular arrays with first-through third-nearest neighbor separations of 1.3, 3.0, and 3.9 cm, respectively. Fibers are kept perpendicular and pressed inward toward the scalp by rigid spacers and rubber stripping on the outside of the cap. To improve coupling of the fibers with the scalp, the fiber tips extend ∼7  mm through the interior of the cap to facilitate combing of the fibers through the hair. Lastly, the cap is held in place against the head with hook-and-loop straps across the forehead of the subject.

### 2.3 Superpixel DOT Acquisition and Algorithm

To successfully use the Andor sCMOS for HD-DOT neuroimaging, the system utilizes spatial and temporal encoding, noise reduction algorithms, and superpixel summing. Individual camera images are 2560 pixels wide by 175 pixels tall [Fig. 3(a)]. This asymmetric region of interest is used to permit faster frame rates. Sets of 25 images (24 source positions and one dark frame) are collected at 6 Hz resulting in a camera frame rate of 150 Hz. The image exposure length is, therefore, 6.7 ms and the laser exposure time with a 75% duty cycle is 5 ms. The light collected by the detector fiber is spread across the CMOS chip as a 31-pixel diameter circle [Fig. 3(b)]. The superpixel algorithm converts individual pixel counts to source–detector pair light levels.

### 2.4 Subjects and Stimulus Protocol

The research was approved by the Human Research Protection Office of the Washington University School of Medicine, and informed consent was obtained from all participants before scanning. We recruited five healthy adult subjects (four females and one male). Subjects sat in an adjustable chair facing a 19-in. liquid crystal display at a 90-cm viewing distance.^3^ We positioned the SP-DOT imaging cap such that the optode array was centered circumferentially on the back of the head with the center of the bottom row of fibers on the inion.

All presented visual stimuli were radial, reversing, black-and-white grids (10-Hz reversal) on a 50% gray background. The paradigm consisted of an angularly swept radial grid wedge that rotated at 10  deg/s to complete a sweep of the entire visual field every 36 s. The grid rotated clockwise three times per stimulus run. The stimulus began with the grid at the bottom of the screen, resulting in a left visual field stimulus at 10 s and a right visual field stimulus at 35 s accounting for the hemodynamic delay. Gray screens were presented for the 30 s before and 15 s after the complete sweep sequence to allow for the visual cortex to return to baseline. The total length of one run with three wedge rotations was, therefore, just under 3 min.

### 2.5 Functional Data Analysis

Changes in absorption coefficient were calculated by normalizing each source–detector pair measurement by their mean and log transformation. The SNR in the heart rate frequency band was calculated as the average power in the pulse frequencies (0.5 to 1.5 Hz) normalized by the total power in the higher frequency noise band (2 to 3 Hz). The first-nearest neighbor (1-nn) pulse SNR per source or detector is calculated as the mean pulse SNR of all the 1-nn measurements of that particular source or detector.

Log-ratio measurement channels were bandpass filtered to 0.008 to 0.2 Hz. Superficial and systemic hemodynamic artifacts were removed by regressing the averaged 1-nn measurements from all other measurements. First, second, and third nearest-neighbor measurements that exhibited a standard deviation <2.5% of the mean signal were used for the image reconstruction. Volumetric reconstructions of absorption coefficients at 830 nm were obtained using a single sensitivity matrix based on the segmented head model of the MNI atlas.^12^^,^^13^ Sensitivity matrices were inverted using a Tikhonov regularization constant of λ=0.01 and a spatially variant regularization parameter of β=0.1.^2^

## 3 Results

### 3.1 Superpixel DOT Algorithm and Specifications

The superpixel algorithm leverages the millions of pixels on a single CMOS chip to increase the DNR and decrease the effective detectivity. The full set of specifications for SP detection is shown in Table 1.

### 3.2 Superpixel DOT Data Quality

**Fig. 5:**
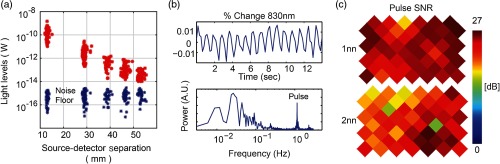
SP-DOT data quality. (a) Data from a healthy volunteer (red measurements) show the expected log-linear decay of light levels with source–detector distance. The spread in the nearest neighbor pairs is less than two orders of magnitude, indicating that there is good coupling between the scalp and all fibers. The first, second, and third nearest-neighbor pairs have SNR>20  dB based on the intensity values compared with the noise floor (blue measurements). (b) Qualitatively, individual detector channels have high enough SNR to show the pulse waveform in the time domain and the frequency domain at ∼1  Hz. (c) A map of this pulse SNR for all source and detector fibers ensures that all fibers are well coupled to the head with SNR>10  dB.

The SP-DOT system and cap produce high-quality data, as measured by a light fall-off curve and high SNR calculated from pulse physiology. The light fall-off curve, which measures the source–detector measurement light levels as a function of their distance, demonstrates the desired log-linear fall off, characteristic of light propagation through biological tissue [Fig. 5(a)]. A cap that does not couple the fibers well to the head would have a shallow, flatter fall-off, and larger variations within similar source–detector separations.

Each individual detector has sufficient SNR to measure the pulse waveform in both the temporal and frequency domains [Fig. 5(b)]. The SNR of this physiological signal for each detector should have an SNR greater than the experimentally derived threshold of 10 dB. We calculate the SNR by dividing the maximum power in the pulse frequencies by the average power in the higher frequency noise. These values, plotted for each source and detector for both first- and second-nearest neighbors, demonstrate a satisfactory cap-fit [Fig. 5(c)].

### 3.3 Visual Activations

**Fig. 6:**
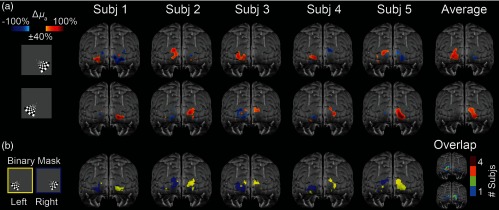
*In-vivo* retinotopic mapping. Subjects viewed a rotating, wedge-shaped checkerboard to stimulate the visual cortex. (a) Left and right block-averaged (n=3 blocks) visual activations can be separately resolved in all five subjects and in the group average (n=5 subjects). (b) Binary mask of the voxels with a value above 40% of the maximum. The overlap map shows the number of subjects with an activated voxel for either the left or right activation, showing moderate overlap incidence between subjects.

For the quadrant-arranged visual stimulations, the images acquired with the SP-DOT system demonstrate neural activations that are both temporally and spatially resolved. The left and right visual cortices are spatially resolved in all participants (n=5) by block averaging only three stimulus repetitions per subject [Fig. 6(a)]. Note that all activations are flipped up-down and left-right due to the anatomy of the visual system. When the stimulus is in the bottom right hand corner of the visual field, a positive change in the absorption coefficient is seen in the left visual cortex [Fig. 6(a), top row]. Similarly, when the stimulus is in the bottom left-hand corner of the visual field, a positive change is seen in the right visual cortex [Fig. 6(a), bottom row]. All activation volumes are normalized to their respective maximum values and thresholded to only show values >40% of the maximum value.

Binary masks of the voxels with a change >40% show symmetrical activations from left and right stimuli [Fig. 6(b)]. Similar spatial results are seen in all five subjects with intersubject variability, as seen with previous HD-DOT systems.^3^ Although an incidence map of the activation overlap shows variability across individual subjects, the activations are generally colocalized [Fig. 6(b)]. Intersubject comparisons might be improved by ensuring consistent cap placement with better cap design and acquiring subject-specific coregistration of the data to anatomical images. The localization differences measured could also be due to variability in the functional architecture of each subject’s brain.^2^^,^^13^^,^^14^

## 4 Discussion

Our results show that the superpixel approach to detection using sCMOS cameras can reduce the weight of HD-DOT imaging arrays by >30-fold compared with standard APD-based HD-DOT systems. Although groups have previously developed CCD-based DOT systems,^15^^–^^18^ these systems have either been too slow to record hemodynamic activity (frame rate <0.01  Hz) or they have been used in geometries that required only limited DNR, such as small volumes (e.g., mouse^15^^,^^18^^–^^20^) or transmission mode measurements^15^^,^^21^ that are very limited for human neuroimaging applications. HD-DOT systems that have had success measuring human neuronal activity using near-infrared wavelengths have all used discrete detectors.^8^ This superpixel approach leverages a monolithic imaging sensor and uses a combination of pixel summing, electronic noise reduction, and spatiotemporal encoding to obtain high DNR ($\mathrm{DNR}>1\times 10^{5}$) and low effective detectivity ($D_{\mathrm{eff}}∼200\;\mathrm{fW}/\surd \mathrm{Hz}/{\mathrm{mm}}^{2}$) at a high frame rate (>6  Hz) using 200-μm-diameter fibers. In addition, our current retinotopic imaging cap weighs only 0.16 lbs including 1 meter of fiber length, ∼30 times lighter than standard imaging caps used in APD-based HD-DOT systems with the same number of source–detector pairs.

Our results show that the SP-DOT imaging system and cap provide high quality *in-vivo* retinotopy activations across multiple subjects. Requiring only three stimulus blocks to produce block-averaged maps in five subjects proves the utility of the SP-DOT system. The structure of the SP-DOT imaging cap is designed to both improve data quality and prevent discomfort by pre-shaping the cap to match the typical head curvature, poking the fiber tips through the cap to comb through the hair, and enabling easy cap placement with hook and loop fastenings.

The reported SP-DOT system demonstrates the potential that sCMOS-based HD-DOT has on improving fiber-based DOT methods. A benefit of using CMOS cameras over other detectors such as APDs is that CMOS cameras can be significantly cheaper. The Andor Zyla sCMOS camera used in the SP-DOT system costs ∼$10,000 but due to economic demand, primarily due to their use in cellphone cameras, their cost is decreasing and their performance is improving.^22^ Demand for higher image resolution and smaller cellphones drives the miniaturization of these sensors. Better picture quality including higher DNR for low-light images drives the sensitivity and performance of the sensors. All of these specification improvements will benefit cell phone cameras, as well as scientific systems like the SP-DOT system.

Fiberless systems have been developed by placing the source and essential detector components directly on the subject’s head. Although these fiberless caps may be more wearable due to a lack of optical fibers, the systems reported thus far have very few measurement channels in the range of 6 to 86.^23^^–^^26^ The superpixel system outperforms those systems by having 264 measurement channels, which translates into higher spatial resolution and/or a larger field-of-view. In the future, it is possible that fiberless systems can be scaled up to whole-head coverage, but thus far this capability has not been shown.

There are several natural extensions to this prototype system, including multiple wavelengths for spectroscopy and larger channel counts for larger field of view. To measure changes in oxy-hemoglobin concentration instead of only the absorption coefficient, an additional wavelength must be used.^27^ The diameter of the right angle housing is wide enough to accommodate two 200-μm-diameter fibers. Each fiber could deliver one wavelength. Using a second wavelength will only increase the effective detectivity by a factor of 2 [Eq. (10)]. To increase the field of view of the current system, more source and detector fibers must be added with appropriate encoding and decoding. Similar to previously published HD-DOT systems,^2^ the encoding and decoding design of the current SP-DOT system allows for the easy expansion of the field of view. The temporal encoding structure of the 24 fibers situated in rectangular panels of four fibers by six fibers separates the groups enough such that each group can operate simultaneously without crosstalk. Adding more sources to a cap, therefore, will not slow down the acquisition time and overall frame rate of the collected data. Similarly, the groups of detector fibers imaged onto the sCMOS camera are separated on the chip enough to prevent crosstalk between two panels. The size of a single image (2560  pixels×175  pixels) can accommodate up to 111 detector fibers per camera. A sCMOS-based superpixel system using lasers can, therefore, accommodate 111 detectors and ∼120 dual-wavelength sources with an effective detectivity below $100\;\mathrm{fW}/\surd \mathrm{Hz}/{\mathrm{mm}}^{2}$.

An HD-DOT system would need approximately 96 sources and 92 detectors for achieving a field-of-view large enough for mapping language processing areas of the brain.^2^ To create a SP-DOT system with this configuration, the console will only require two detector boxes (7 RU or 12 in. height) and 6 RU (10.5 in. height) worth of source boxes. Including a computer, source, and detector components, along with encoding and synchronization hardware, such a system would only take up $∼12\;{\mathrm{ft}}^{2}$ (4-ft height×19-in. width×2-ft depth) compared with $∼44\;{\mathrm{ft}}^{2}$ required for a 96 source and 92 detector APD-based system (two racks each 7-ft height×19-in. width×2-ft depth).^2^ The superpixel sCMOS approach allows for a fourfold reduction in HD-DOT system size compared with APD-based systems without sacrificing system resolution. Additionally, a whole-head cap with 96 detector fibers and 184 dual-wavelength source fibers would weigh only 0.64 lb.

Combined with HD-DOT image quality, the portability and wearability of the SP-DOT system could significantly improve studies requiring a naturalistic environment.^28^ In-person social interactions could be investigated while the subjects sit comfortably on a couch. SP-DOT could also be used in the clinic for continuous, longitudinal imaging of hospital patients while they lie in their bed, which could impact clinical care decisions and improve patient outcomes. In addition, SP-DOT could enhance applications to newborn and childhood medicine including monitoring in neonatal intensive care units and cooperation from toddlers to study their neural development.^29^

## Appendix A: Calculating the NEP of a Single Pixel

The minimum power required to achieve an SNR of 1 can be related to the photoelectrons generated by the pixel. First, we express the optical power in terms of photoelectrons

$$P_{0}=\frac{hc\phi }{\lambda }=\frac{hc}{\lambda }(\frac{Q}{t\eta_{\lambda }}),$$ (13)

where $P_{0}$ is the optical power, h is the Planck’s constant, ϕ is the photon flux in units of photons/sec, λ is the wavelength of the photon, Q is the number of photoelectrons generated, t is the exposure time, and $\eta_{\lambda }$ is the quantum efficiency at wavelength λ. Plugging in Eq. (13) into Eq. (2), the NEP can be defined as a function of photoelectrons

$$\mathrm{NEP}=\frac{Qhc}{\lambda t\eta_{\lambda }\sqrt{BW}}.$$ (14)

To derive an expression for the NEP of a single pixel, we need to determine the number of photoelectrons required to achieve an SNR of 1. For a cooled CMOS sensor, the primary noise sources are shot noise, dark noise, and read-out noise

$$\mathrm{SNR}=\frac{Q}{\sqrt{Q+i_{d}t+N_{r}^{2}}},$$ (15)

where $i_{d}$ is the dark current, t is the integration time, and $N_{r}$ is the rms read-out noise. By setting the SNR equal to one and assuming the shot noise and dark current are negligible at low light levels, we arrive at an expression for the photoelectrons required for an SNR of 1

$$Q=N_{r}.$$ (16)

Finally, the NEP at 1 Hz of a single pixel is given by

$$\mathrm{NEP}=\frac{N_{r}hc}{\lambda \eta_{\lambda }}.$$ (17)

The Zyla 5.5 sCMOS has an rms read-out noise of 2.5 electrons and quantum efficiency of 27.5% at 830 nm, which results in an NEP of 0.0022  fW/√Hz. If N pixels are summed, then the variance of the readout noise increases by a factor of N, and the number of photoelectrons required per pixel for achieving an SNR of 1 increases by $\sqrt{N}$.

## Appendix B: Comparing Commercially Available APD Modules

We compared six commercially available APD modules: Hamamatsu C12703, Hamamatsu C12703-01, Excilitas 902-200, Excilitas 954-200, and the Laser Components S500-10. The module with the lowest specification NEP is the C12703-01 3-mm-diameter APD module. Consequently, this module also has the lowest effective detectivity using 200-μm-diameter fibers [Table 2, Eq. (12), temporal encoding steps K=16, duty cycle d=0.5, and 50% packing fraction]. Custom module designs using 200-μm-diameter APDs and cooling may be possible but at the moment has not been realized in a commercially available product.

**Table 2:** APD module comparison. The Hamamatsu C12703-01 detector has the lowest effective detectivity of commercially available modules.

	Diameter (mm)	NEP (fW/√Hz)	D ($\mathrm{fW}/{\mathrm{mm}}^{2}$)	$D_{\mathrm{eff}}$ ($\mathrm{fW}/{\mathrm{mm}}^{2}/\surd \mathrm{Hz}$)
Excilitas 902-200	0.5	42	1338	15,133
Excilitas 954-200	0.5	110	3503	39,634
Laser Components S500-10	0.5	30	955	10,809
Hamamatsu C12703	1.5	200	6369	72,062
Hamamatsu C12703-01	3	20	637	7202
